# Pandemic influenza A/H1N1 virus infection and *TNF*, *LTA*, *IL1B*, *IL6*, *IL8*, and *CCL* polymorphisms in Mexican population: a case–control study

**DOI:** 10.1186/1471-2334-12-299

**Published:** 2012-11-13

**Authors:** Guadalupe Morales-García, Ramcés Falfán-Valencia, Román Alejandro García-Ramírez, Ángel Camarena, Alejandra Ramirez-Venegas, Manuel Castillejos-López, Martha Pérez-Rodríguez, César González-Bonilla, Concepción Grajales-Muñíz, Víctor Borja-Aburto, Juan Manuel Mejía-Aranguré

**Affiliations:** 1 Coordinación de Investigación en Salud, Instituto Mexicano del Seguro Social (IMSS), Torre Academia Nacional de Medicina 4to piso, Av. Cuauhtémoc 330, 06720 México, DF, México; 2 Instituto Nacional de Enfermedades Respiratorias Ismael Cosío Villegas, Tlalpan 4502, 14080, México, DF, México; 3 Coordinación de Vigilancia Epidemiológica y Apoyo en Contingencias, IMSS, Mier y Pesado 120, 03100, México, DF, México

**Keywords:** TNF, IL1B, IL8, IL6, LTA, CCL1, Influenza AH1N1

## Abstract

**Background:**

Some patients have a greater response to viral infection than do others having a similar level of viral replication. Hypercytokinemia is the principal immunopathological mechanism that contributes to a severer clinical course in cases of influenza A/H1N1. The benefit produced, or damage caused, by these cytokines in severe disease is not known. The genes that code for these molecules are polymorphic and certain alleles have been associated with susceptibility to various diseases. The objective of the present study was to determine whether there was an association between polymorphisms of *TNF*, *LTA*, *IL1B*, *IL6*, *IL8*, and *CCL1* and the infection and severity of the illness caused by the pandemic A/H1N1 in Mexico in 2009.

**Methods:**

Case–control study. The cases were patients confirmed with real time PCR with infection by the A/H1N1 pandemic virus. The controls were patients with infection like to influenza and non-familial healthy contacts of the patients with influenza. Medical history and outcome of the disease was registered. The DNA samples were genotyped for polymorphisms *TNF* rs361525, rs1800629, and rs1800750; *LTA* rs909253; *IL1B* rs16944; *IL6* rs1818879; *IL8* rs4073; and *CCL1* rs2282691. Odds ratio (OR) and the 95% confidence interval (95% CI) were calculated. The logistic regression model was adjusted by age and severity of the illness in cases.

**Results:**

Infection with the pandemic A/H1N1 virus was associated with the following genotypes: *TNF* rs361525 AA, OR = 27.00; 95% CI = 3.07–1248.77); *LTA* rs909253 AG (OR = 4.33, 95% CI = 1.82–10.32); *TNF* rs1800750 AA (OR = 4.33, 95% CI = 1.48–12.64); additionally, *LTA* rs909253 AG showed a limited statistically significant association with mortality (p = 0.06, OR = 3.13). Carriers of the *TNF* rs1800629 GA genotype were associated with high levels of blood urea nitrogen (p = 0.05); those of the *TNF* rs1800750 AA genotype, with high levels of creatine phosphokinase (p=0.05). The *IL1B* rs16944 AA genotype was associated with an elevated number of leukocytes (p <0.001) and the *IL8* rs4073 AA genotype, with a higher value for P_a_O_2_ mm Hg.

**Conclusion:**

The polymorphisms of genes involved in the inflammatory process contributed to the severity of the clinical behavior of infection by the pandemic influenza A/H1N1 virus.

## Background

The influenza A/H1N1 virus pandemic of 2009 started in Mexico and then spread worldwide, with an alert level of pandemic phase 6 declared by the World Health Organization (WHO) in June of that year [1]. Although the majority of those infected by the influenza A/H1N1 virus presented mild symptoms that were self-limiting, a subgroup of patients followed an adverse clinical course, thus requiring a greater level of medical attention and more aggressive management [2]. In Mexico the lethal rate was estimated to be 1.2% for cases of influenza-like illness (ILI) and 5% for confirmed cases of influenza A/H1N1 [3]. Some co-morbidities (e.g., immunosuppression, pre-existing pulmonary disease, cardiac disease, diabetes, asthma that requires regular medical attention, smoking, and obesity) have been demonstrated to increase the risk of hospitalization for infection with influenza A/H1N1 [4,5]. The risk is also augmented in the second and third trimesters of pregnancy and when treatment with oseltamivir is prescribed five days after the onset of the illness [6]. In addition, the results of certain laboratory tests (e.g., lactate dehydrogenase (LDH), >600 IU/L; hypoxemia (P_a_O_2_, <60 mm Hg); C-reactive protein (CRP), 10 mg/dl; and leukopenia <5000/mm^3^) have been associated with greater mortality from infection by influenza A/H1N1 virus [7,8].

Through experimental and clinical studies, it has been determined that the most important pathological mechanism in this infection is systemic dysregulation of the inflammatory response, which is correlated with the severity and progression of the illness [9,10]. The secretion of cytokines by infected cells appears to be necessary for the initiation of the immunological response that controls the replication of the virus [11]; in addition, the presence of immunopathological mechanisms, such as hypercytokinemia (“cytokine storm”), generally is considered to contribute to the severest evolution of the infection [11-13]. Elevated levels of pro-inflammatory cytokines and chemokines (e.g., TNFα, IFNγ, IL-1, IL-6, IL-8, IL-9, IL-12 IL-15, and IL-17) have been found, up to ten days after the onset of symptoms, in the plasma of patients with acute respiratory distress syndrome (ARDS) caused by influenza A/H1N1 [9,10,14]. The genes that code for these molecules are polymorphic and certain alleles have been associated with susceptibility to various diseases that cover a wide range of pathologies, from infectious to oncological, including pulmonary and systemic diseases [15-30]. The role that the polymorphisms of the genes encoding these cytokines play in the severity of the disease is not clear.

The extensive polymorphism of these molecules may be associated with the high mortality rate during the 2009 influenza A/H1N1 pandemic in Mexico. Because we think that genetic factors of the host may influence the nature and intensity of the inflammatory immune response, the objective of this study was to determine whether the polymorphisms of genes that are associated with inflammation may be associated with the development of the infection and with the clinical severity in Mexican mestizo patients with influenza A/H1N1.

## Methods

The rapid QuickVue Influenza A+B test (Quidel, San Diego, CA, USA) was used to analyze the nasopharyngeal swab samples obtained from the patients in 94 cases of suspected infection by influenza A/H1N1, following the recommendations for collection and testing of the U.S. Centers for Disease Control and Prevention (CDC) and of the WHO [31,32]. The patients were then separated into two groups: those positive for influenza A/H1N1 (A/H1N1 group) and those negative, as having influenza-like illness (ILI group). A total of 44 patients were positive for the influenza A/H1N1 virus (A/H1N1 group); the remaining 50 patients who had tested negative for the influenza A/H1N1 virus, were diagnosed as having influenza-like illness (ILI).

In addition, a group of 176 asymptomatic healthy contacts (AHC group) voluntarily participated in the study. Those in the AHC group, although not biologically related to any patient, were in personal contact with at least one of the patients during the period of the illness. To ensure that those in the AHC group had been exposed to the virus, their titers of anti-influenza A/H1N1 antibodies were determined. To evaluate the presence of antibody, we use haemagglutination inhibition technique (HAI); contacts exhibited significant titers of specific anti-A/H1N1 antibodies, supporting the fact that they were in contact with the A/H1N1 virus. By serially diluted aliquots of serum samples; those individuals with titers greater than 1:16 were considered positive for A/H1N1 infection/exposure. The two patient groups and the AHC group were Mexican mestizos (age range: 18–85 years). All patients suspected of influenza A/H1N1 infection were treated with anti-viral therapy (oseltamivir) upon admittance at the hospital.

The information collected in this study included demographic, clinical history, laboratory test data, pharmacological treatment, and follow-up. This study was approved by the Institutional Committee of Science and Bioethics of the National Institute of Respiratory Diseases (code B05-10). The study protocol was explained to all participants and signed informed consent was duly obtained from each participant. This information was obtained by means of a clinical form in accord with Official Mexican Standards Mexicana NOM-168-SSA1-1998; topics covered included age; gender; tobacco smoking; body-mass index (BMI; patients with BMI >30 k/m^2^); and disease morbidity (pulmonary, hepatic, renal, cardiac, neurological diseases, diabetes mellitus, hypertension, and cancer). The symptoms evaluated were fever, cough, rhinorrhea, dyspnea, nasal congestion, thoracic pain, headache, diarrhea, and vomiting. The begin of anti-viral therapy was evaluated in relation to the days with previous symptomatology. The laboratory parameters included were leukocyte titer; lactate dehydrogenase (LDH); creatine phosphokinase (CPK); blood urea nitrogen (BUN); and arterial gases (with P_a_O_2_ <60 mm Hg defined as severe disease). Pneumonia was verified by radiological findings. All patients admitted to the intensive care unit (ICU) and those put on assisted mechanical ventilation (AMV) were identified.

### Genotyping of allelic variants **(**single nucleotide polymorphism, SNPs)

The DNA samples were genotyped for polymorphisms *TNF* rs361525, rs1800629, and rs1800750; *LTA* rs909253; *IL1B* rs16944; *IL6* rs1818879; *IL8* rs4073; and *CCL1* rs2282691 using Taqman commercial probes (Apliedd Biosystems, USA) the primers listed in Table 1. In brief, the procedure for the real time PCR was the following: 15 ng DNA; 15 μL of Taqman universal PCR master mix (Roche NJ, USA) and 6.5 μL of each probes. The conditions for amplification were the following: 94°C (3 min), 61°C (1 min), and 72°C (1 min); followed by 35 cycles of 94°C (1 min), 61°C (1 min), and 72°C (1 min,); and a final cycle of 94°C (1 min), 61°C (1 min), and 72°C (5 min). The genetic data for the SNPs were analyzed and are listed in Table 2.

**Table 1:** DNA samples were genotyped by using Taqman commercial probes

**Gene (reference SNP**)	**Probe**
*TNF* (rs1800750)	GAGGCAATAAGACCCCCCTCGGAATC[A/G]GAGCAGCTGTCAATTGCAGGAGCT
*TNF* (rs1800629)	GAGGCAATAGGTTTTGAGGGGCATG[A/G]GGACGGGGTTCAGCCTCCAGGGTCC
*TNF* (rs361525)	GGCCCAGAAGACCCCCCTCGGAATC[A/G]GAGCAGGGAGGATGGGGAGTGTGA
*LTA* (rs909253)	AAGCCTTAAAACCTAGGGCATACA[C/T]TTGATAATTCACCCTCCAGGGTCCGTT
*IL1B* (rs16944)	TACCTTGGGTGCTGTTCTCTGCCTC[G/A]GGAGCTCTCTGTCAATTGCAGGAGC
*IL6* (rs1818879)	AGACGAGCTGGGCGCAGTGGCTCAC[A/G]CCTATAATCCCAGCACTTTGGGAGG
*IL8* (rs4073)	TTATCTAGAAATAAAAAAGCATACA[A/T]TTGATAATTCACCAAATTGTGGAGC
*CCL1* (rs2282691)	AAAAAGCCTTAAAATACTGACTGGT[A/T]TGTGAAAGCTACTCCAATTAAGTTT

*SNP* Single nucleotide polymorphisms.

**Table 2:** Genetic data of the single nucleotide polymorphisms (SNPs) analyzed

**SNP**	**Gene**			
	**Symbol**	**Location**	**Position**	**Alleles**
rs1800750	*TNF*	−376	Promoter	G/A
rs1800629	“	−308	Promoter	G/A
rs361525	“	−238	Promoter	G/A
rs909253	*LTA*	+252	Intronic	C/T
rs16944	*IL1B*	−511	Promoter	G/A
rs1818879	*IL6*	5845	3^′^UTR	A/G
rs4073	*IL8*	−251	Promoter	A/T
rs2282691	*CCL1*	29712422	Intronic	A/T

### Analysis

When the polymorphisms were evaluated, the ancestral genotype was used for comparison. The odds ratio (OR) and the 95% confidence interval (95% CI) were calculated. In the logistic regression model, the OR was adjusted by age and severity of the illness. The χ^2^ test was used to evaluate the differences between the proportions of the groups. The differences among the clinical parameters, continuous variables, and polymorphisms were evaluated by using the Mann–Whitney *U* test. Software packages SPSS 19 (IBM, Chicago, IL) and Epi-Info 6.04b were used (Atlanta, CDC).

## Results

The patients were separated into two groups depending whether they were positive or negative for influenza A/H1N1, thereby forming the groups, influenza A/H1N1 and ILI, respectively. In both the A/H1N1 and ILI groups, the majority was male (68.0% and 58.0%, respectively), whereas in the AHC group, 61.93% were female. The mean age of the A/H1N1 and ILI groups was <45 years (68.18% and 60.0%, respectively), compared to that of the AHC group, 56.82% of who were 45–64 years of age.

The data concerning the demographics, co-morbidities, and symptomatology for both groups of patients are presented in Table 3. Interestingly, the mortality was higher for patients with infection by influenza A/H1N1 than for those with ILI (34.09% *vs*. 4.0%, respectively; P<0.001); similarly, hospitalization in the ICU was more frequent for the influenza A/H1N1 group (54.55% vs. 30.0%, respectively; p = 0.01). No statistically significant differences were found for any of the other variables analyzed. Of the influenza A/H1N1 patients, 93.3% had radiographic signs of pulmonary compromise, with a statistically significant difference between the two groups of patients (p = 0.043; data not shown).

**Table 3:** Demographical and clinical characteristics of influenza A/H1N1 patients, influenza-like illness (ILI) patients, and healthy control subjects

**Characteristic**	**A/H1N1 patients**	**ILI patients**	**Healthy controls**	**p**
	**(Total: 44)**	**(Total: 50)**	**(Total: 176)**	
	**n (%)**	**n (%)**	**n (%)**	
**Sex**				
Male	30 (68.18)	29 (58.0)	67 (38.07)	
Female	14 (31.82)	21 (42.0)	109 (61.93)	
**Age**				
<45	30 (68.18)	30 (60.0)	54 (30.68)	
45-64	11 (25.00)	13 (26.0)	100 (56.82)	
≥65	3 (6.82)	7 (14.0)	22 (12.50)	
**BMI ≥30**	18 (40.91)	21 (42.0)	46 (26.42)	
**Mortality**	15 (34.09)	2 (4.0)		<0.001*
**P**_**a**_**O**_**2**_**<60 mm Hg (severe)**	19 (43.18)	24 (48.0)		
**ICU**	24 (54.55)	15 (30.0)		0.01*
**Co-morbidities**				
Neurological disease	25 (56.82)	22 (44.0)		
Asthma	2 (4.55)	6 (12.0)		
Cancer	1 (2.27)	2 (4.0)		
Hypertension	8 (18.18)	7 (14.0)		
Smoking	21 (47.73)	29 (58.0)		
**Symptomatology**				
Fever (>38°C)	33 (75.00)	42 (84.0)		
Cough	23 (52.27)	31 (62.0)		
Nasal Congestion	5 (11.36)	2 (4.0)		
Rhinorrhea	12 (27.27)	20 (40.0)		
Dyspnea	33 (75.00)	35 (70.0)		

*When comparing A/H1N1 patients *vs.* ILI patients. BMI: Body mass index; P_a_O_2_: partial pressure of oxygen in arterial blood; ICU: Intensive Care Unit. Results were considered statistically significant when P was <0.05.

### Analysis of genetic association

Genotyping was carried out for eight SNPs from six genes, the protein products of which have been associated with inflammatory processes. The genetic information of the polymorphisms that were evaluated is presented in Table 4. An appreciation of the genetic contribution to the risk of infection by influenza A/H1N1 was obtained by evaluating genotype and alleles of both patient groups and comparing the frequencies of the genotypes and alleles with those of the AHC group.

**Table 4:** Genetic frequency of the genotypes of eight SNPs in six genes and their association with infection by influenza A/H1N1 virus

**Gene and genotype**	**Genetic frequency**								
	**A/H1N1 group**	**AHC group**	**p**	**OR**	**95% CI**	**ILI group**	**p**	**OR**	**95% CI**
*CCL1* rs2282691									
AA	0.643	0.402	0.0085	2.67	1.26-5.81	0.630	0.0096	2.53	1.23-5.30
TA	0.286	0.456				0.304			
TT	0.071	0.142				0.065			
*IL1B* rs16944									
GA	0.535	0.449				0.583			
GG	0.442	0.364				0.375			
AA	0.023	0.188	0.015	0.10	0.00-0.66	0.042	0.024	0.19	0.02-0.79
*IL8* rs4073									
AT	0.659	0.445	0.0231	2.40	1.12-5.32	0.500			
AA	0.244	0.396				0.386			
TT	0.098	0.159				0.114			
*IL6* rs1818879									
GG	0.439	0.268				0.381			
AG	0.390	0.530				0.452			
AA	0.171	0.202				0.167			
*LTA* rs909253									
CT	0.535	0.244	0.0004	3.56	1.68-7.54	0.540	0.00014	3.63	1.79-7.38
TT	0.302	0.488	0.0432	0.45	0.20-0.97	0.320	0.0517*	0.49	0.24-1.00
CC	0.163	0.267				0.140			
*TNF*									
rs361525									
GG	0.818	0.884				0.894			
AA	0.136	0.006	0.00031	27.0	3.07-1248.77	0.000			
GA	0.045	0.110				0.106			
rs1800629									
GG	0.932	0.946				0.911			
GA	0.068	0.054				0.089			
AA	0.000	0.000							
rs1800750									
GG	0.789	0.949	0.0035	0.20	0.06-0.66	0.830	0.0116	0.26	0.08-0.84
AA	0.158	0.028	0.005	6.41	1.51-27.92	0.128	0.0128	5.00	1.20-21.61
GA	0.053	0.023				0.043			

A/H1N1 group: patient infected with influenza A/H1N1 virus; AHC group: asymptomatic, healthy contacts; ILI group: patients with influenze-like illness; OR: odds ratio; CI: confidence interval. Results were considered statistically significant when p was <0.05.

For the A/H1N1 and AHC groups, of the eight SNPs used, 24 genotype products were generated; in the ILI group, only 23 genotypes were determined, as the genotype AA does not exist for the rs361525 of the gene *TNF*. For the A/H1N1 group, five genotypes associated with risk were identified (p <0.05; OR >2.0). Of particular interest was the finding of homozygous A genotype in SNPs rs361525 and rs1800750 in *TNF*, both with values of OR >5.0. In addition, the genotypes rs2282691 AA, rs4073 AT, and rs909253 CT (*CCL1*, *IL8*, and *LTA*, respectively) demonstrated statistically significant association with risk. For the ILI group, three of the five associations previously reported for the A/H1N1 group were found. It is of note that these associations, which coincided in both patient groups, showed statistically significant data (p values and OR) that were very similar. On the other hand, our findings demonstrated the existence of three genotypes with association to protection (p <0.05; OR ≤1.0): *IL1B* rs16944 AA, *LTA* rs909253, and *TNF* rs1800750 GG, in both patient groups, as compared to the AHC group (Table 4).

In the analysis of alleles, two signs that showed statistically significant association with risk were found: allele A of rs2282691 of *CCL1* was shown to be increased in both patient groups (A/H1N1, p <0.05; ILI, p <0.01), when the allelic frequency (AF) of each patient group was compared with that of the AHC group (OR = 2.15 and 2.11, respectively). Also, rs1800750 allele A, one of the three polymorphisms evaluated for *TNF*, showed a similar behavior, that is, an association with risk in both patient groups (A/H1N1, p <0.01; ILI, p <0.01). No other association was found for the remaining six SNPs analyzed (Table 5).

**Table 5:** Risk of influenza A/H1N1 infection in relation to the different polymorphisms studied

**Gen/allele**	**Risk of influenza A/H1N1 infection**		
	**p**	**OR (95% CI)**	**OR allelic (95% CI)**
*CCL1*			2.31 (1.25-4.31)
rs2282691			
AT	0.85	1.14 (0.29-4.38)	
AA	0.12	2.75 (0.78-9.72)	
*IL1B*			1.62 (0.92-2.88)
rs16944			
GG	0.04	8.57 (1.11-66.46)	
AG	0.06	7.33 (0.95-56.41)	
*IL8*			1.45 (0.80-2.63)
rs4073			
TT	0.96	0.97 (0.29-3.28)	
AT	0.17	1.73 (0.79-3.74)	
*IL6*			1.01 (0.59-1.71)
r1818879			
AG	0.2	0.61 (0.29-1.30)	
AA	0.45	0.69 (0.26-1.82)	
*LTA*			0.72 (0.42-1.26)
rs909253			
GG	0.66	1.25 (0.46-3.41)	
AG	<0.001	3.3 (1.51-7.20)	
*TNF*			1.25 (0.79-1.99)
rs1800750			
AG	0.27	2.54 (0.49-13.24)	
AA	0.02	3.52 (1.24-10.03)	
rs1800629			1.10 (0.30-3.91)
AG	0.88	1.1 (0.30-4.02)	
rs361525			3.34 (1.56-7.08)
AG	0.37	0.5 (0.11-2.24)	
AA	<0.001	34.8 (4.06-297.87)	

OR: odds ratio; CI: confidence interval; allelic OR: comparison with ancestral gene. Results were considered statistically significant when P was <0.05.

### Association of genotypes with clinical variables

#### Mortality

The genotype AG of rs909253 in *LTA* showed a tendency to be associated with mortality (OR = 3.13), with p = 0.06 just above the limit of statistical significance. Table 6 presents the data for the different genotypes evaluated with respect to mortality. The data corresponding to the allelic OR were carried out for both alleles; however, only those carried out for the ancestral allele are shown. No data for the remaining alleles or genotypes studied were statistically significant. The logistic regression analysis, after adjusting for levels of P_a_O_2_ and for admission to the ICU, showed no statistically significant association between the genotypes and mortality.

**Table 6:** Risk of death from influenza A/H1N1 in relation to the different polymorphisms studied

**Gen/allele**	**p**	**OR (CI 95%)**	**OR allelic (CI 95%)**
*CCL1* rs2282691			1.63 (0.63-4.40)
AT	0.40	0.4 (0.07-2.79)	
AA	0.90	1.1 (0.22-5.31)	
*IL1B* rs16944			1.55 (0.56-4.50)
GG	0.70	1.6 (0.17-14.40)	
AG	0.30	2.8 (0.35-23.02)	
*IL8* rs4073			1.41 (0.53-3.78)
TT	0.70	0.7 (0.07-6.11)	
AT	0.40	1.7 (0.51-5.76)	
*IL6* rs1818879			0.96 (0.40-2.28)
AG	0.30	0.5 (0.15-1.70)	
AA	0.50	0.5 (0.10-2.76)	
*LTA* rs909253			0.46 (0.16-1.27)
GG	0.50	0.5 (0.05-4.27)	
AG	*0.06*	*3.1 (0.93-10.53)*	
*TNF*			
rs1800750			1.21 (0.55-2.61)
AG	0.30	3.5 (0.39-31.76)	
AA	0.20	2.9 (0.58-14.51)	
rs1800629			2.39 (0.51-11.03)
AG	0.30	2.5 (0.51-12.26)	
rs361525			0.95 (0.21-4.16)
AG	1.00		
AA	0.40	2.8 (0.31-24.74)	

OR: odds ratio; CI: confidence intervals; allelic OR: comparison with ancestral gene. Results were considered statistically significant when P was <0.05.

#### Polymorphisms and laboratory tests

No changes in laboratory parameters were found with respect to the genotypes of the SNPs in *TNF* rs361525, *LTA* rs909253, *CCL1* rs2282691, or *IL6* rs1818879 ( Additional file 1: Table S1). For the homozygous genotype AA of rs16944 in *IL1B*, an elevated number of leukocytes was found (mean: 44.9 × 10^3^/mm^3^), whereas for the heterozygous genotype AG and the homozygous genotype GG, the mean titer values were 8.62 ×10^3^/mm^3^ and 6.96 ×10^3^/mm^3^, respectively (p <0.001). The genotype AG in rs1800629 in *TNF* was associated with elevated levels of BUN (33mg/dl), whereas for the genotype GG, the BUN levels were lower (p =0.05) (Additional file 1: Table S1). The genotype AA of rs1800750 in *TNF* showed high levels of CPK (mean: 2246.25 IU/L), whereas the genotypes GG and AG had mean values of 366.32 IU/L and 450.00 IU/L, respectively (p=0.05).

## Discussion

Although it has been reported that some of the genes involved in inflammation are associated with pulmonary and infectious diseases [16,17,21,25-28], to date, no direct association between these polymorphisms and infection by influenza A/H1N1 virus has been reported. In the present study, the logistic regression analysis of cases and controls showed that *TNF* rs361525 (AA), *rs1800750* (AA), and *LTA* rs909253 (AG) were associated with high risk of infection by pandemic influenza A/H1N1. Although mortality of the A/H1N1 patients was greater than that of the ILI patients (p <0.001), only genotype AG of *LTA* rs909253 demonstrated an association with mortality (p = 0.06) just missing being statistically significant. This finding may indicate that being a carrier of genotype AG LTA at rs909253 entails a poorer prognosis for this illness.

Some biomarkers, such as CRP, LDH, leukopenia, and hypoxemia [7,8] have been considered as predictors for severe illness by influenza A/H1N1 virus. In this study, we found that elevated values of laboratory test parameters (BUN, CPK, and leukocyte titer) were associated with *TNF* (rs1800629 AG, rs1800750 AA), and *IL1B* (genotype AA). These results may indicate that being a carrier of these polymorphisms in particular may be associated with severer organic damage by infection by influenza A/H1N1 virus. However, in the case of *IL8*, the homozygous AA appeared to offer a certain degree of protection against severe illness; this may be explained, in part, by the association of a higher concentration of oxygen in the blood (P_a_O_2_ >60 mm Hg) with this genotype.

Although susceptibility for presenting an adverse clinical course (i.e., sepsis, septic shock, multiples organ failure, or death) varies due to different degrees of inflammatory response [30], genetic factors of the host may influence the nature and intensity of this response. The present study is the first to demonstrate that the polymorphisms in genes related to the inflammatory response may, in some manner, be influencing the risk of infection by influenza A/H1N1 virus and of death from this illness. One possible mechanism may be the formation of disequilibria in the bond between alleles, creating haplotypes that differentially affect the expression and activity of cytokines and chemokines, thereby resulting in a severer clinical course for the infection.

To avoid variability in our results, we studied a homogeneous population of Mexican mestizos. Although the sampling size was a limitation in our study, we were able to demonstrate statistically significant differences in the distribution of genotypes in terms of infection, mortality, and biomarkers between cases and controls, thus suggesting a strong association with the illness. However, infection by influenza A/H1N1 virus is a very complex illness that involves not only the association of environmental factors and the genetic make-up and biology of the individual per se, but also the presence of co-morbidities that may contribute to a greater severity of the illness [4,5].

We had limitations. For example, contacts exhibited significant titers of specific anti-A/H1N1 antibodies, supporting the fact that they were in contact with the A/H1N1 virus. Additionally is necessary clear that is not a patients group, we just include unrelated contacts in this study (e.g. family in law persons, home workers, etc.). They were in close contact with patients when the latter exhibited acute respiratory illness. None of these household contacts developed respiratory illness. However, the presence of antibody would not confirm infection with A/H1N1 virus because there is the likelihood of cross-reactivity [33]. But, when a person with positivity of antibodies that had contact with an A/H1N1 virus infected patient, the infection cannot be excluded [34]. The gold standard for identifying the infection to A/H1N1 virus is real time PCR test; however to identify the presence of the virus in symptomatic patients is necessary that the patients be assessed during the first days from the beginning of the disease, the probability of identifying the virus by molecular test in asymptomatic patients is very low and not practical [35].

With the high mortality rate from the 2009 influenza A/H1N1 pandemic in Mexico [31], the approach used in this work could acquire great importance, as the study of polymorphisms may be useful in predicting the conduct that the infection would follow during future outbreaks of influenza A/H1N1 in Mexico.

## Conclusions

The *TNF* polymorphisms studied were associated with risk of infection by influenza A/H1N1 virus during the pandemic in Mexico in 2009. These genetic variants may contribute to the severest clinical manifestations in Mexican mestizos.

## Abbreviations

AHC: Asymptomatic healthy contacts; ARDS: Acute respiratory distress syndrome; BMI: Body mass index; BUN: Blood urea nitrogen; CCL1: Chemokine (C-C motif) ligand 1; CDC, U.S: Centers for Disease Control and Prevention; CI: 95% Confidence interval; CPK: Creatine phosphokinase; CRP: C- reactive protein; GLR: Global lethal rate; ICU: Intensive Care Unit; IFN: Interferon; IL: Interleukin; ILI: Influenza-like illness; LDH: Lactate dehydrogenase; LTA: Lymphotoxin α; OR: Odds ratio; PaO_2_: Partial pressure of oxygen in arterial blood; SNP: Single nucleotide polymorphism; TNF: Tumor necrosis factor; WHO: World Health Organization.

## Misc

Guadalupe Morales-García and Román Alejandro García-Ramírez these authors contributed equally to the development of this manuscript

## Competing interests

The authors declare that they have no conflict of interests.

## Authors’ contributions

GMG, RFV, RAGR, AC, and MCL carried out the study and participated in its design and co-ordination, in the molecular assays, and in the preparation of the manuscript. ARV participated in the design and co-ordination of the study, compiled data, and helped prepare the manuscript. MPR, CGB, CGM, VBA, and JMMA participated in the design and co-ordination of the study, carried out the study and the statistical analysis, and helped prepare the manuscript. All authors read and approved the final version of the manuscript.

## Pre-publication history

The pre-publication history for this paper can be accessed here:

http://www.biomedcentral.com/1471-2334/12/299/prepub

## Supplementary Material

Additional file 1 **Table S1.** Comparison of laboratory findings in relation to the polymorphisms studied in A/H1N1 patients and controls. Click here for file
