# Supplementary material for: Pandemic influenza A/H1N1 virus infection and TNF, LTA, IL1B, IL6, IL8, and CCL polymorphisms in Mexican population: a case–control study
Source: BMC Infect Dis. 2012 Nov 13;12:299. doi: 10.1186/1471-2334-12-299 (PMC3534505; doi:10.1186/1471-2334-12-299)
Supplement: Additional file 1 — Table S1. Comparison of laboratory findings in relation to the polymorphisms studied in A/H1N1 patients and controls. [file 1471-2334-12-299-S1.doc]

**Supplemental Information Table 1. Comparison of laboratory findings in relation to the polymorphisms studied in A/H1N1 patients and controls.**

| **Group** | **Polymorphism** | **Laboratory test** | | | | |
| --- | --- | --- | --- | --- | --- | --- |
|  |  | **Leukocyte titer** | **BUN** | **CPK** | **LDH** | **P_a_O_2_** |
|  |  | **Mean (s.d)** | **Mean (s.d)** | **Mean (s.d)** | **Mean (s.d)** | **Mean (s.d)** |
| **Alive** |  | 8576 (4313.299) | 14.05 (11.493) | 340.25 (333.357) | 622.03 (274.669) | 61.43 (22.566) |
| **Dead** |  | 13366.67 (15711.609) | 16.33 (10.912) | 2200.75 (3403.144) | 889.17 (344.215) | 67.72 (27.369) |
|  | **P** | 0.053 | 0.642 | 0.000 | 0.028 | 0.521 |
|  | **TNF238** |  |  |  |  |  |
| Controls | AG | 9125 (3050) | 12.75 (5.500) | 248 (88.544) | 495.75 (84.255) | 78.05 (23.221) |
|  | AA | ND | ND | ND | ND | ND |
|  | GG | 8505.56 (3831.445) | 15.52 (13.412) | 338.40 (361.925) | 692.83 (336.744) | 60 (17.735) |
|  | **P** | 0.751 | 0.689 | 0.625 | 0.256 | 0.085 |
| Patients | AG | 7050 (3323.405) | 6 (1.414) | 598 (541.644) | 585 (2.828) | 57 (5.515) |
|  | AA | 7150 (4158.926) | 10 (2.828) | 440.50 (323.224) | 823.67 (416.505) | 74.50 (64.630) |
|  | GG | 9315.38(8063.334) | 14 (10.786) | 716 (1468.585) | 671.87 (277.705) | 58 (20.563) |
|  | **P** | 0.818 | 0.454 | 0.93 | 0.613 | 0.591 |
|  | **LTA252** |  |  |  |  |  |
| Controls | AA | 9333.33 (3203.049) | 13.31 (5.907) | 311.21 (359.850) | 624.40 (321.349) | 73.15 (21.805) |
|  | GG | 6940 (3471.023) | 14.50 (4.435) | 287.80 (135.345) | 798.60 (249.469) | 61.56 (23.600) |
|  | GA | 8818.18 (4794.107) | 15.91 (16.056) | 339.68 (362.513) | 657.86 (342.487) | 57.16 (12.425) |
|  | **P** | 0.542 | 0.843 | 0.942 | 0.588 | 0.041 |
| Patients | TT | 12,966.67 (12637.148) | 13.75 (15.031) | 335.50 (396.303) | 622.33 (272.812) | 66.54 (21.906) |
|  | CC | 5883.33 (2336.165) | 10.67 (4.041) | 619.17 (425.997) | 825.25 (306.892) | 71.08 (56.198) |
|  | CT | 7817.65 (3388.627) | 14.43 (8.140) | 868.47 (1789.187) | 679 (284.017) | 54.13 (18.555) |
|  | **P** | 0.132 | 0.857 | 0.633 | 0.5 | 396 |
|  | **TNF308** |  |  |  |  |  |
| Controls | AG | 10175 (2942.080) | 14.33 (5.132) | 159.50 (83.688) | 562.20 (224.176) | 65.63 (21.189) |
|  | GG | 8450 (3896.949) | 15.47 (13.681) | 364.45 (362.574) | 700.53 (337.282) | 62.68 (19.016) |
|  | **P** | 0.399 | 0.889 | 0.273 | 0.433 | 0.774 |
|  | **H1N1** |  |  |  |  |  |
| Patients | AG | 6900 (1555.636) | 33 (ND) | 320 (ND) | 689 (48.083) | 51.10 (.141) |
|  | GG | 9036.67 (7658.598) | 12.96 (9.612) | 682.36 (1336.082) | 681.04 (290.872) | 61.36 (29.171) |
|  | **P** | 0.701 | 0.053 | 0.792 | 0.97 | 0.628 |
|  | **TNF376** |  |  |  |  |  |
| Controls | AG | 11950 (7990.307) | 47.50 (50.205) | 249 (169.706) | 610.50 (81.317) | 60.55 (10.536) |
|  | AA | 7516.67 (2890.963) | 10.83 (1.722) | 206.80 (149.450) | 787.50 (288.996) | 64.83 (18.192) |
|  | GG | 8553.13 (3627.581) | 13.90 (6.236) | 353.25 (371.660) | 655.59 (341.232) | 62.47 (19.688) |
|  | **P** | 0.356 | 0.000 | 0.651 | 0.647 | 0.95 |
| Patients | AG | 9700 (ND) | ND | 450 (ND) | 567 (ND) | 36.10 (ND) |
|  | AA | 8940 (4407.720) | 10.60 (6.189) | 2246.25 (3346.055) | 859.50 (414.987) | 57.84 (26.367) |
|  | GG | 9523.81 (8695.626) | 14.88 (11.810) | 366.32 (342.766) | 610.40 (214.798) | 61.37 (17.578) |
|  | **P** | 0.989 | 0.45 | 0.051 | 0.208 | 0.45 |
|  | **IL1B** |  |  |  |  |  |
| Controls | AA | 5900 (ND) | 8 (ND) | 69 (ND) | 417 (ND) | 55 (ND) |
|  | GG | 8013.33 (2999.968) | 14.07 (6.592) | 468.53 (427.584) | 773.40 (317.940) | 60.20 (14.000) |
|  | AG | 9449 (4731.455) | 16.04 (15.423) | 215.54 (192.775) | 576.76 (280.807) | 65.98 (21.950) |
|  | **P** | 0.457 | 0.774 | 0.4 | 0.105 | 0.617 |
| Patients | AA | 44900 (ND) | 7 (ND) | ND | 804 (ND) | 94.30 (ND) |
|  | GG | 6957.14 (3654.652) | 16.25 (15.040) | 394.50 (334.919) | 675.54 (264.765) | 67.48 (37.122) |
|  | AG | 8618.75 (3372.085) | 13.33 (7.480) | 923 (1856.614) | 685.21 (320.625) | 53.04 (16.844) |
|  | **P** | 0.000 | 0.658 | 0.304 | 0.916 | 0.211 |
|  | **IL8** |  |  |  |  |  |
| Controls | AA | 7385.71 (3192.023) | 12.25 (4.351) | 365.69 (409.722) | 779.14 (346.618) | 61.07 (15.002) |
|  | TT | 7966.67 (1497.776) | 11.50 (2.121) | 541.33 (776.681) | 478.33 (158.658) | 72.10 (22.170) |
|  | AT | 9261.90 (4261.042) | 17.19 (16.473) | 285.57 (224.817) | 642.29 (313.418) | 66.78 (21.825) |
|  | **P** | 0.356 | 0.55 | 0.472 | 0.256 | 0.573 |
| Patients | AA | 12044.44 (12810.163) | 12 (6.066) | 1441.29 (2572.947) | 701.71 (362.765) | 78.37 (41.130) |
|  | TT | 8900 (2685.144) | 12.50 (0.707) | 345.33 (197.224) | 560.67 (153.050) | 49.07 (6.278) |
|  | AT | 7100 (3625.725) | 15.60 (12.374) | 459.35 (427.970) | 697.06 (285.261) | 54.37 (18.515) |
|  | **P** | 0.295 | 0.764 | 0.252 | 0.755 | 0.098 |
|  | **CCL1B** |  |  |  |  |  |
| Controls | AT | 9269.23 (3525.711) | 12.08 (2.968) | 298 (424.603) | 554.54 (306.098) | 75.27 (25.480) |
|  | AA | 7591.30 (3369.909) | 13.57 (6.954) | 306.18 (225.517) | 763.30 (335.220) | 57.99 (14.219) |
|  | TT | 9666.67 (4235.957) | 16.67 (4.163) | 649 (675.826) | 559.33 (184.058) | 55.73 (6.133) |
|  | **P** | 0.308 | 0.458 | 0.261 | 0.15 | 0.034 |
|  | AT | 7766.67 (3650.685) | 9.80 (5.495) | 363.33 (320.582) | 693.13 (279.766) | 65.11 (43.027) |
|  | AA | 10020 (8888.881) | 13.94 (10.831) | 772.56 (1645.161) | 660.94 (294.618) | 61.07 (20.510) |
|  | TT | 6350 (2333.452) | 20 (18.385) | 981 (ND) | 621 (48.083) | 52.10 (1.556) |
|  | **P** | 0.663 | 0.507 | 0.744 | 0.938 | 0.84 |
|  | **IL6** |  |  |  |  |  |
| Controls | AG | 7731.25 (3709.037) | 13.81 (4.564) | 358.13 (364.052) | 639.38 (289.589) | 57.07 (14.237) |
|  | AA | 9580 (4175.763) | 10.25 (2.872) | 154.20 (131.351) | 422.20 (96.422) | 88.40 (27.826) |
|  | GG | 8750 (3143.430) | 14.33 (7.548) | 378 (421.287) | 791.50 (374.393) | 60.51 (15.649) |
|  | **P** | 0.541 | 0.467 | 0.492 | 0.082 | 0.005 |
| Patients | AG | 8176.92 (3567.715) | 13.18 (9.336) | 279.33 (286.907) | 740.54 (225.894) | 54.45 (19.760) |
|  | AA | 7600 (1663.330) | 11 (1.414) | 324.25 (113.837) | 615.25 (128.126) | 44.83 (7.517) |
|  | GG | 9914.29 (10822.687) | 15.64 (12.258) | 1213.50 (1938.114) | 645 (381.234) | 71.28 (36.256) |
|  | **P** | 0.794 | 0.788 | 0.198 | 0.639 | 0.172 |

BUN: blood urea nitrogen; CPK: creatine phosphokinase; LDH: lactate dehydrogenase; P_a_O_2_ : partial pressure of oxygen in arterial blood s.d.: standard deviation; ND: No Data; results were considered statistically significant when P was <0.05.
